# *Mycobacterium indicus pranii* Supernatant Induces Apoptotic Cell Death in Mouse Peritoneal Macrophages *In Vitro*

**DOI:** 10.1371/journal.pone.0017093

**Published:** 2011-02-11

**Authors:** Rajeev Kumar Pandey, Kunal H. Bhatt, Yogesh Dahiya, Ajit Sodhi

**Affiliations:** School of Biotechnology, Faculty of Science, Banaras Hindu University, Varanasi, India; University of Hyderabad, India

## Abstract

*Mycobacterium indicus pranii* (MIP), also known as Mw, is a saprophytic, non-pathogenic strain of *Mycobacterium* and is commercially available as a heat-killed vaccine for leprosy and recently tuberculosis (TB) as part of MDT. In this study we provide evidence that cell-free supernatant collected from original MIP suspension induces rapid and enhanced apoptosis in mouse peritoneal macrophages *in vitro*. It is demonstrated that the MIP cell-free supernatant induced apoptosis is mitochondria-mediated and caspase independent and involves mitochondrial translocation of Bax and subsequent release of AIF and cytochrome c from the mitochondria. Experiments with pharmacological inhibitors suggest a possible role of PKC in mitochondria-mediated apoptosis of macrophages.

## Introduction

*Mycobacterium indicus pranii* (MIP) is a non-pathogenic, saprophytic and cultivable strain of *Mycobacterium* and belongs to Runyon group IV based on its growth and biochemical characteristics [1]. A vaccine against leprosy based on MIP is approved for human use, where it has resulted in modest clinical improvement, accelerated bacillary clearance, and increased immune responses to *Mycobacterium leprae* antigens, thereby shortening the full recovery time of the patients [2], [3], [4]. Recently MIP has emerged as a broad spectrum vaccine candidate as it shares antigens not only with *M. leprae* but also with *M. tuberculosis*. Recently conducted clinical trials involving approximately 30,000 human subjects have shown that vaccination with heat killed MIP induced protection against TB and also resulted in early sputum conversion in TB patients [5]. Studies with animal models have shown that MIP, commercially available as “Immuvac” confers protection against *M. tuberculosis* infection in both BCG responder and non-responder genetic strains of mice [6], [7]. The ongoing polyphasic molecular and biochemical studies have assigned a discrete & unique phylogenetic position to MIP [8]. MIP does not cause infection in any of the animal models in which it has been tested inspite of its close resemblance to *Mycobacterium avium* complex; based on the initial results of the clinical trials, it is under extensive trial for a number of serious diseases including cancer [7], [9]-[12]. Owing to these unique immunologic properties, MIP appears to be a promising & safe philanthropic vaccine candidate.

During the early stages of infection with intracellular pathogens like *M. tuberculosis*, the extent of bacterial survival and proliferation is mainly determined by the efficacy of the innate immune response with macrophages as the main effector cells [13], [14]. To look for immunomodulatory properties of heat killed MIP, effect of MIP on murine peritoneal macrophages was investigated *in vitro*. Macrophages were treated with increasing volumes of the original commercial suspension. While lower volumes led to macrophage activation (unpublished data), rapid cell death of macrophages was observed at higher volumes. The experiments with inserts revealed that induced cell death of macrophages was independent of the direct contact between the heat-killed bacterium & the macrophage cell surface (data not shown). To resolve the case further, the study was conducted using either the cell-free supernatant (as defined in ‘materials and methods’ section) or washed cell pellet alone. While the washed cell pellet of heat-killed MIP led to differential activation of macrophages (unpublished data), interestingly, the cell-free supernatant was found to induce rapid cell death of mouse peritoneal macrophages *in vitro*. Based upon a number of independent parameters we present evidence that MIP cell-free supernatant induces programmed cell death of mouse peritoneal macrophages *in vitro* which is caspase independent and involves induction of the mitochondrial pathway. We also demonstrate that at lower doses the cell free supernatant leads to a significant downregulation of LPS-induced proinflammatory cytokine expression in peritoneal macrophages *in vitro*. We propose that the strong apoptogenic activity of MIP supernatant may reduce the potency of MIP as a vaccine & may in part be responsible for the early side-effects at the site of injection.

## Materials and Methods

### Ethics Statement

The studies presented in this manuscript were approved by the Scrutiny Committee of the School of Biotechnology, Banaras Hindu University, as per the University directive No. R/Dev/Project 1987/dt. 31-11-1987.

### Mice

Inbred strains of Balb/c mice of either sex at 8–10 weeks of age were used for obtaining peritoneal macrophages.

### Cell cultures and reagents

Macrophages were cultured in RPMI 1640 medium supplemented with heat-inactivated fetal calf serum (10%), Penicillin (100 U/ml), Streptomycin (100 U/ml) and Gentamycin (20 ug/ml) at 37°C in humidified air containing 5% CO_2_. Heat-killed MIP suspension, thiomersal were obtained from Cadila Pharmaceuticals, India. Proteinase K, Polymixin B, Medium RPMI 1640, TRI-reagent, Staurosporin, Rottlerin, H7, Annexin V-Cy3 APOAC apoptosis detection kit, caspase-3 & caspase-8 fluorimetric assay kits and most of the other reagents, until mentioned otherwise, were obtained from Sigma-Aldrich Chemicals, St Louis, MO, USA. Fetal calf serum was purchased from Biological Industries, Israel. Broad spectrum caspase inhibitor Z-VAD-fmk, caspase-3 inhibitor Ac-DEVD-CHO and caspase 8 inhibitor Z-IETD-fmk were purchased from Imgenex Co, San Diego, CA, USA. Mitocapture Apoptosis detection kit and Nucleosome ELISA kit were from Calbiochem, La Jolla, CA, USA. Polyclonal antibodies against caspase-3 & 8, Bax, PARP, PKC δ, Actin, AIF and cytochrome c, FITC conjugated anti-Bax, TRITC conjugated anti-AIF were obtained from Santa Cruz Biotechnology Inc, CA, USA. All the reagents were endotoxin-free as determined by the Limulus amoebocyte lysate assay (sensitivity limit, 0.1 ng/ml).

### Preparation of MIP cell-free supernatant

Original heat-killed MIP suspension contains 5×10^9^ MIP cells/ml suspended in 0.9% w/v sodium chloride & 0.01% w/v thiomersal. The original suspension was centrifuged at 10,000 rpm and the cell-free supernatant & pellet were carefully separated under strictly sterile conditions. The pellet containing MIP cells was washed and resuspended to the original volume in sterile PBS. Mouse peritoneal macrophages were treated either with different volumes (60–100 ul/ml/10^6^ cells) of the cell-free supernatant (henceforth termed MIP supernatant) or with resuspended MIP cell pellet for different time intervals. Doses mentioned as sub-threshold are ≤40 ul/ml/10^6^ cells/well.

### Macrophage Isolation and cytotoxicity assay

Macrophage monolayers were prepared as described previously [15]. Peritoneal exudate cells (PECs) were harvested from the healthy, inbred strain of Balb/c mice (8–10 weeks old, 20–22gms) using chilled serum-free RPMI 1640 medium and plated in the wells of 24 well plate (Nunc, Denmark). After 2 hr incubation at 37°C in an atmosphere of 5% CO_2_ in a CO_2_ incubator, the non-adherent cells were removed by washing with warm serum-free medium and the adherent cells were further incubated in complete medium overnight to form macrophage monolayer. More than 95% of the adherent cell population was macrophages as determined by morphology and non-specific esterase staining.

Cytotoxic activity of MIP supernatant was measured by MTT (4, 5-dimethylthiazol-2-yl-2, 5-diphenyl tetrazolium bromide) assay [16]. Briefly peritoneal macrophages (10^6^ cells/well) were cultured in 24 well culture plates in 1 ml RPMI 1640 medium. After overnight culture, the cells were washed and then treated with various doses of MIP supernatant for different time intervals in fresh medium. To exclude the possibility of cytotoxicity by MIP supernatant due to endotoxin contamination, macrophages were incubated with polymyxin B (20 µg/ml)-treated MIP supernatant for 8 hr. After completion of treatment, 50 µl of 5 mg/ml MTT solution was added to the monolayers and incubated for 4 hr at 37°C. The MTT reaction was terminated by the addition of 0.04N HCl in isopropanol. The MTT formazan formed was measured spectrophotometrically (540 nm) using an EMax Spectrophotometer (Molecular Devices, Sunnyvale, CA, USA).

The % cytotoxicity was calculated by formula:
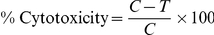



Where C =  absorbance ‘control’ represents macrophages incubated in medium alone and T =  absorbance ‘experimental’ represents macrophages treated with MIP supernatant.

### Immunofluorescence staining

Peritoneal macrophage monolayers on cover glass were treated with MIP supernatant for 4–6 hr. To evaluate nuclear morphology, the cells on cover glass were fixed with 4% paraformaldehyde, stained with DAPI (10 µg/ml) in phosphate-buffered saline (PBS) for 10 min, and then observed under fluorescence microscope (Olympus BX61, Olympus optical Co. Ltd. Tokyo, Japan).

For staining Bax or AIF, macrophage monolayers on cover glass were treated with MIP supernatant for 1–4 hr. After treatment, macrophage monolayers were washed with warm incomplete medium. The cells were then fixed in 4% paraformaldehyde for 10 min at room temperature, washed (three times) in PBS and permeabilized with 0.1% Triton X-100 for 5 min at room temperature. Cells were washed twice in PBS and incubated in PBS containing 1% BSA for 60 min, blocking the nonspecific binding sites. Subsequently, the cells were incubated with anti-Bax-FITC or anti-AIF-TRITC antibody overnight at 4°C in dark, washed with chilled PBS containing 0.05% Tween 20 (three times), observed and photographed with Olympus fluorescence microscope. Non-specific fluorescence staining was ruled out using non-specific isotypic antibodies (data not shown).

### Apoptosis Assays

Annexin V-Cy3 and Mitocapture Apoptosis detection kits were used to detect apoptosis in macrophages under fluorescence microscope. For Annexin V-Cy3 staining, macrophage monolayers on cover-glass were incubated for 4 hr with MIP supernatant (100 ul/ml/10^6^ cells). The cells were washed twice with PBS and then with 1× binding buffer (3 times), stained with Double Label Staining Solution (AnnCy3+6-CFDA) as per manufacturer's instructions. After 10 min, the monolayers were washed with 1× binding buffer (5 times) and observed under fluorescence microscope. The green fluorescing cells were scored as healthy cells, whereas green cells with red periphery were considered apoptotic. Untreated cells served as control.

The MitoCapture Apoptosis Detection Kit was used for detection of mitochondrial membrane depolarization (Δψ_m_). Untreated or MIP supernatant treated macrophage monolayers were incubated with 1 µg/ml of Mitocapture cationic dye at 37°C for 20 min. Cells were photographed using fluorescence microscope with excitation wavelengths of 500 and 570 nm. Cells exhibiting green fluorescence indicating lowering of mitochondrial transmembrane potential were counted as apoptotic cells. Cells with red specks for mitochondria indicate presence of J-aggregates, and thus were considered as normal cells. At least 5 independent spots were observed for each slide.

### Quantification of oligonucleosome units resulting due to DNA fragmentation

The Nucleosome ELISA Kit was used to quantify free oligonucleosome units resulting due to MIP supernatant-induced DNA fragmentation in macrophages as recommended by the manufacturer. Briefly, macrophage monolayers treated with MIP supernatant were washed and lysed. The lysates were used to measure free oligonucleosome units by antigen capture ELISA. The optical density at 450 nm was read in EMax Spectrophotometer (Molecular Devices, Sunnyvale, CA, USA). Triplicate wells were assayed for each condition. Free oligonucleosome DNA fragments were quantified according to the manufacturer's instruction manual.

### Caspase activity assays

The caspase-3 and caspase-8 activities were determined by using fluorimetric assay kits. Briefly, macrophages were pretreated either with caspase-8 inhibitor Z-IETD-FMK (50 µM) or caspase-3 inhibitor Ac-DEVD-CHO (100 µM) for an hour in incomplete medium. The medium was replaced and macrophages were incubated in fresh complete medium with MIP supernatant for 2 hr. The cells were then lysed as per the instructions provided. 5 µl of cell lysates were added to the wells of a 96 well flat bottom plate. For blank 5 µl of 1X assay buffer was used. To some wells 200 µM of caspase-3 or caspase-8 inhibitor was added. 200 µl of reaction mixture was then added gently to each well. Plate was covered for 12 hr in dark (kept at −80°C) and read at 360 nm excitation and 460 nm emission at 37°C in a Microplate Fluorescence Reader (FLx 800, Bio-Tek Instruments, Inc., USA).

### Preparation of cell lysates and immunoblotting

The macrophages with or without treatment were washed with ice cold PBS containing 1 mM Na_3_VO_4_, then lysed in 50 µl of RIPA lysis buffer supplemented with protease inhibitor cocktail for 20 min at 4°C. The lysates were centrifuged (13,000 g at 4°C) for 15 min and the supernatant (containing Triton X-100 soluble proteins) were separated on 10% SDS-polyacrylamide gels at 20 mA. The separated proteins were transferred onto nitrocellulose membrane (45 min. at 200 mA) using Bio-Rad mini transblotter and immunoblotted with primary antibody, incubated with secondary antibody conjugated with horseradish peroxidase and visualized by the chemiluminescence Western blotting kit (Santa Cruz Biotechnology, CA, USA). Cytosolic extract (excluding nuclei and mitochondria) was prepared using a previously reported method and used for western blotting against cytochrome c antibody. For nuclear translocation studies, purified nuclei were isolated using NE-PER nuclei extraction kit (Pierce, Rockford, IL, USA). To monitor equal loading of protein, western blot analysis using antibody directed against Actin was done for each experiment as shown in lower panels.

### Enzyme Linked Immunosorbent Assay (ELISA)

Macrophage monolayers were treated with specified doses of MIP supernatant/& other reagents as mentioned, supernatant was collected & ELISA was performed as per manufacturer's instruction.

### MALDI-Tof analysis of the ∼46 kDa supernatant band

MIP cell-free supernatant was lyophilized and re-suspended in sterile PBS & run on SDS-PAGE. The protein band visible at ∼46 kDa was cut & sent for MALDI-Tof analysis which was performed by ILS Bioservices Pvt Ltd, Gurgaon, India.

### Statistical Analysis

The statistical significance of difference between the test groups was analyzed by student's t-test (two tailed). *p*-value of less than 0.05 is considered significant. The error bars of the values represent 95% confidence interval.

## Results

### MIP cell-free supernatant induces apoptosis of peritoneal macrophages *in vitro*

**Figure 1:**
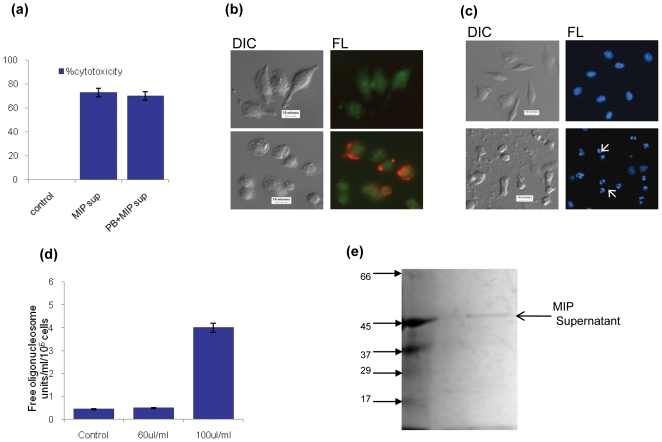
MIP supernatant induces apoptosis in mouse peritoneal macrophages. (a) MIP supernatant induced cell death of macrophages was not due to LPS contamination. Macrophage monolayers were treated with MIP supernatant (1 µg/ml) for 8 hr and % cytotoxicity was determined by MTT assay. Before incubation of macrophages with MIP supernatant, the supernatant was also treated either with Polymyxin B (PB). Untreated cells were taken as control with 0% cytotoxicity. (b) Murine peritoneal macrophages after treatment with MIP supernatant for 4 hr were dual stained with Sigma APOAC kit. Annexin V stained early apoptotic cells (annexin V positive, 6-CFDA positive) show red fluorescence on cell surface. Untreated viable cells (annexin V negative, 6-CFDA positive) fluoresce green with no signal for Annexin V. (c) Macrophage monolayers were treated with MIP supernatant for 6 hr, fixed & stained for DAPI. Arrows show nuclear condensation & fragmentation. Scale bar: 10 µm. (d) Macrophage monolayers were treated with MIP supernatant for 6 hr, lysed and the DNA fragmentation was detected by quantitative Nucleosome ELISA with anti-histone antibody. Untreated cells were taken as control. (e) MIP cell-free supernatant was lyophilized and re-suspended in sterile PBS & run on SDS-PAGE. The gel was stained with AgNo_3_ and observed. Lane1: Low molecular weight SDS marker, lane2: MIP supernatant. The indicated molecular weights are in kDa.

MIP cell-free supernatant resulted in significant cell death of peritoneal macrophages. The percent cytotoxicity of MIP supernatant was quantitatively determined by MTT assay. It was observed that 60–70 ul of MIP supernatant induced cell death in ∼40–45% of macrophages by 8 hr of incubation. However, 100 ul/ml of MIP supernatant resulted into about 70–75% cell death of macrophages in the same time interval (Fig. 1a). To examine if the mode of induced cell death was apoptosis, macrophage monolayers were incubated with a range of supernatant volumes and assessed for detection of Phosphatidylserine exposure on the outer leaflet of the cell membrane by annexin V binding. Significant levels of apoptosis were apparent with as little as 60 ul/ml of MIP supernatant and continued to increase through 100 ul/ml, the maximum dose examined (Fig. 1b). The treatment of macrophage monolayers with MIP supernatant resulted into DNA fragmentation in a dose dependent fashion whereas chromatin condensation and nuclear fragmentation were also observed by DAPI staining at 6 hr of treatment (Fig. 1c, d).

### Proteomic analysis of MIP cell-free supernatant

Cell-free supernatant consistently showed a band of ∼46 kDa on SDS-PAGE (Fig. 1e). MALDI-TOF analysis of the band resulted into a number of fragments which showed a significant homology to Peptide Chain Release Factor 1 of bacterial origin. Following three tryptic fragments showed partial identities to two proteins, namely 1) a putative short chain dehydrogenase of unknown function, and 2) RpoB protein, when BLASTed against the *Mycobacterium indicus pranii* (taxid: 35617) database:

1). SQLQNKERAMQMLR

2). TYNYPQSRVTDHR

3). RTMVATGDRSAK

Interestingly, the first and the second fragments had domain hits for prfA (Peptide Chain Release Factor 1) in the conserved domains database (http://www.ncbi.nlm.nih.gov/Structure/cdd/cdd.shtml).

### MIP cell-free supernatant induced apoptosis is not due to LPS contamination

To examine whether LPS contamination might be contributing to apoptosis induction by MIP supernatant, supernatant was incubated with polymyxin B before addition to macrophage monolayers. The proapoptotic effect of supernatant was found to be polymyxin B- resistant which indicated that LPS contamination was not contributing to the apoptosis observed in cells exposed to supernatant protein (Fig. 1a).

### MIP cell-free supernatant induces cleavage of caspase 3, PARP

**Figure 2:**
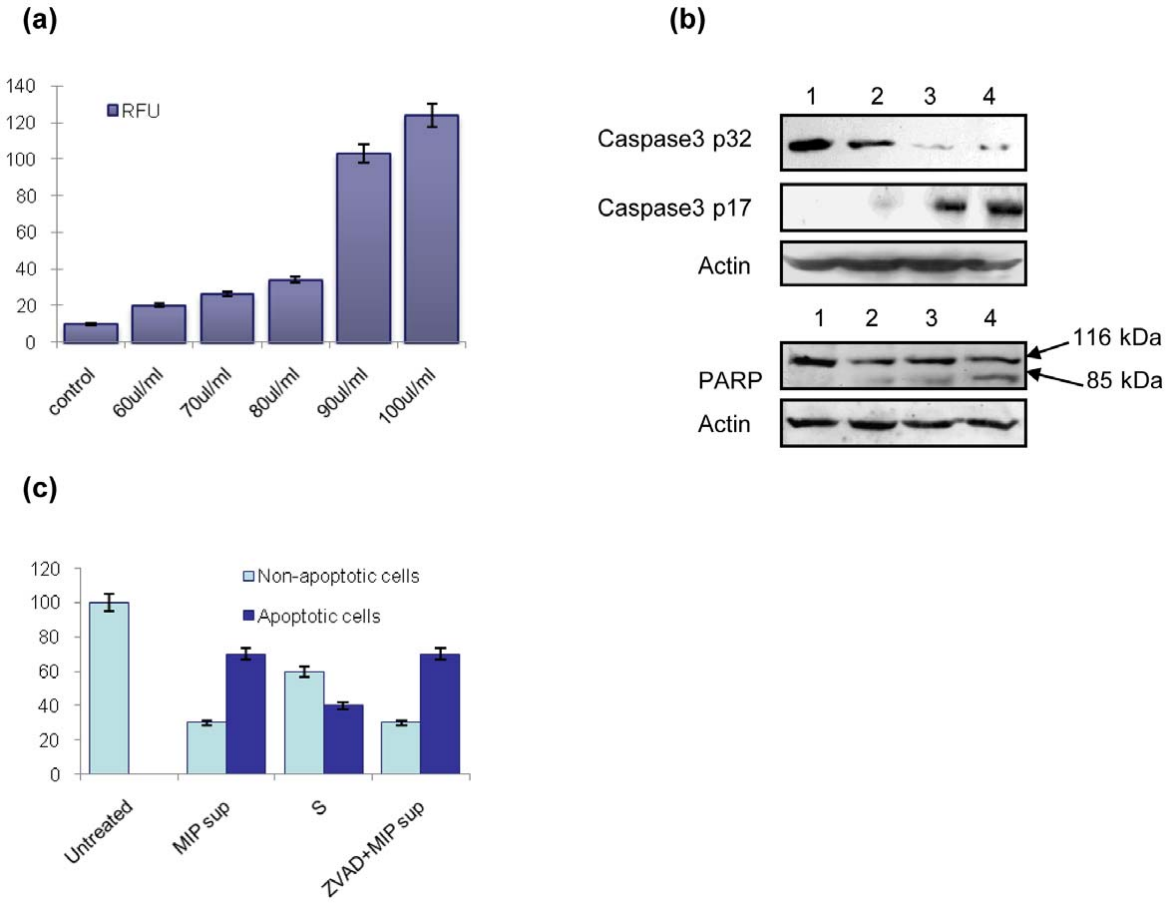
MIP supernatant induced caspase activation and PARP cleavage. (a) Measurement of cleaved (activated) caspase3 in Relative Fluorescence Units (RFU) as a direct evidence for enhanced apoptosis of macrophages on treatment with MIP supernatant (60–100 ul/ml). Macrophage monolayers were treated with different concentrations of MIP supernatant for 90 min & activated caspase3 was quantified using Sigma caspase3 FL detection kit. (b) MIP supernatant induced caspase-3 activation and PARP degradation in peritoneal macrophages. Macrophage monolayers were incubated with MIP supernatant (1 µg/ml) for 1, 2, 4 hr; the cells were harvested and examined by western blotting for procaspase-3 (32 kDa) (upper panel), cleaved caspase-3 (p17) (middle panel) and PARP (116 kDa) degradation into the main proteolytic product of PARP (85 kDa). Anti-actin Ab was used in parallel as a loading control (lower panels). Lane 1: Untreated control, 2: 1 hr, 3: 2 hr, 4: 4 hr. (c) Treatment of murine peritoneal macrophages with MIP supernatant elicited disruption of mitochondrial trans-membrane potential. Mitochondrial membrane potential was visualized with a MitoCapture Mitochondrial Apoptosis Detection kit. Pretreatment with pan-caspase inhibitor Z-VAD-fmk had no inhibitory effect on MIP supernatant induced MMP. Staurosporin (S) (0.5 uM) treated cells were taken as a positive control. Bars in the figure show % of non-apoptotic (red fluorescence) & apoptotic cells (Red & green fluorescence).

Treatment of macrophages with MIP supernatant led to activation of caspase 3. The estimation of caspase 3 activity was based on the ability of the supernatant treated cell lysates to hydrolyze the fluorogenic peptide substrate of caspase 3, acetyl-Asp-Glu-Val-Asp-7-amido-4-methylcoumarin (Ac-DEVD-AMC), resulting in the release of the fluorescent 7-amino-4-methylcoumarin (AMC) moiety which can be quantified. The expression of cleaved active form of caspase 3 (p17) increased with time (Fig. 2a, b). Cleavage of caspase 3 substrate PARP was also observed in macrophages treated with MIP supernatant (Fig. 2b). On the other hand MIP supernatant did not result in the activation of caspase 8.

### MIP cell-free supernatant induced apoptosis is mitochondria mediated

Treatment of macrophages with MIP supernatant led to lowering of mitochondrial membrane potential (MMP) (Δψ_m_). Changed MMP was observed in macrophages using Mitocapture cationic dye. Macrophages showing green fluorescence suggest cells with changed mitochondrial membrane potential, whereas cells with normal mitochondria show red fluorescence. The number of macrophages with altered mitochondrial membrane potential significantly increased with time on treatment with MIP supernatant. By 4 hr of treatment ∼70% of cells showed green fluorescence, and thus decreased Δψ_m_. The green and red fluorescing cells observed under fluorescence microscope were counted from at least five different spots and their mean is expressed as % apoptotic cells. Pretreatment of macrophages with either caspase-8 inhibitor Z-IETD-fmk or pan-caspase inhibitor Z-VAD-fmk had no effect on the onset of mitochondrial membrane potential (Δψ_m_) disruption induced by MIP supernatant (Fig. 2c).

### MIP cell-free supernatant causes release of cytochrome c, AIF from mitochondria and nuclear translocation of AIF

**Figure 3:**
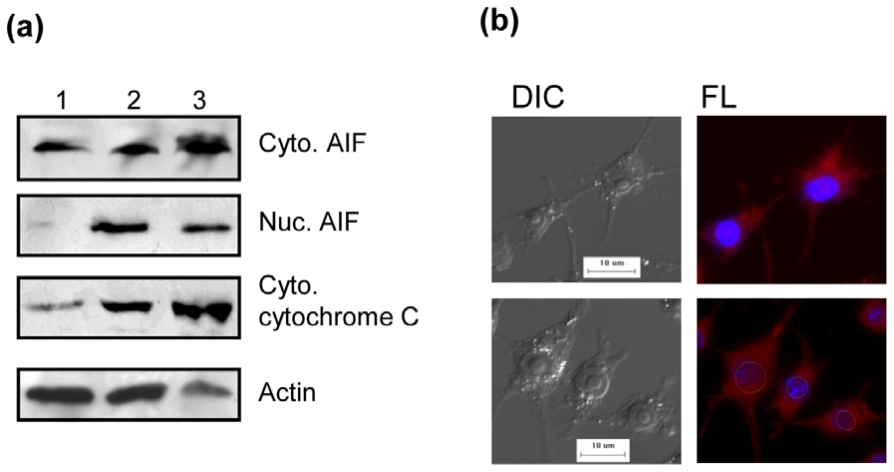
MIP supernatant induced release of AIF and Cytochrome c into the cytoplasm, nuclear translocation of AIF. (a) Macrophage monolayers were incubated with MIP supernatant for 3, 4 hr; the cytoplasmic and nuclear fractions were harvested and examined by western blotting for AIF and cytochrome c. Lane 1: untreated cells, lane 2: 3 hr, lane 3: 4 hr. (b) Macrophage monolayers grown overnight on cover-glasses were treated with MIP supernatant for 4 hr, washed & fixed with 4% paraformaldehyde, probed with anti-AIF-TRITC & Hoechst and visualized for nuclear accumulation of AIF under Olympus BX61 fluorescence microscope. Upper panel: untreated, lower panel: MIP supernatant treated. The areas encircled clearly demonstrate co-localization of a red signal for AIF in the nucleus in MIP supernatant treated macrophages. Scale bar: 10 µm.

Mitochondria mediated apoptosis may involve the release of cytochrome c and AIF from the mitochondria into the cytoplasm. This possibility was tested by fractionating nuclei, cytoplasm and mitochondria from MIP supernatant treated macrophages. Cytoplasmic cytochrome c & AIF and also the nuclear translocation of AIF were analyzed by western blot. Significantly enhanced cytoplasmic expression of cytochrome c & AIF as well as nuclear translocation of AIF was evident after 4 hr of treatment (Fig. 3a). The fluorescence microscopy also demonstrated the nuclear accumulation of AIF 4 hr post-treatment (Fig. 3b).

### Treatment with MIP cell-free supernatant induces Bax translocation to mitochondria

**Figure 4:**
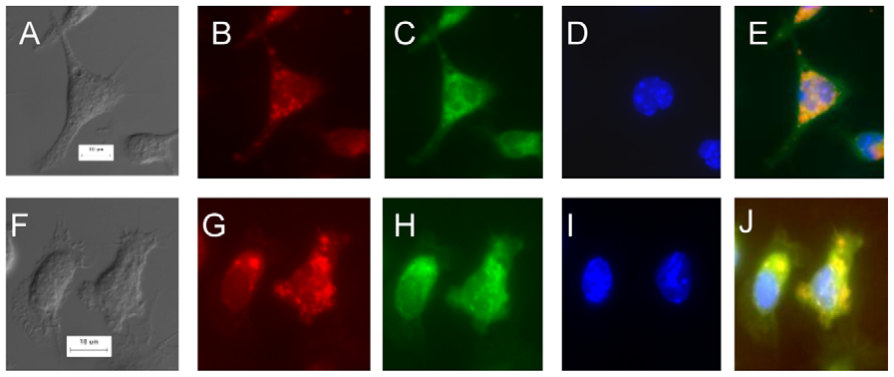
MIP supernatant induces Bax translocation to the mitochondria. Macrophage monolayers were treated with MIP supernatant for 1–1.5 hr and fixed. Cells were probed for Bax-FITC and Hoechst, mitochondria were stained red. The upper panel (A–E) shows untreated macrophages while the lower panel (F–J) displays MIP supernatant treated macrophages. E and J show the overlay of respective panels. It is clear from the overlay that there is distinct overlapping of red & green fluorescence indicating co-localization of Bax with mitochondria in the MIP supernatant treated macrophages (J). Scale bar: 10 µm.

Activation of Bax and its mitochondrial translocation are the key regulatory events during induction of mitochondrial membrane depolarization and result into lowering of Δψ_m_. Treatment of macrophages with MIP supernatant led to mitochondrial translocation of Bax by as early as 1.5-2 hr of treatment. The translocation of Bax-FITC (green fluorescence) to the mitochondria (red fluorescence) is clearly demonstrated by co-localization of green & red fluorescence. On the other hand, in untreated macrophages the endogenous Bax was found diffused throughout the cytoplasm (Fig. 4).

### Inhibition of PKC inhibits MIP cell-free supernatant induced apoptosis in macrophages

**Figure 5:**
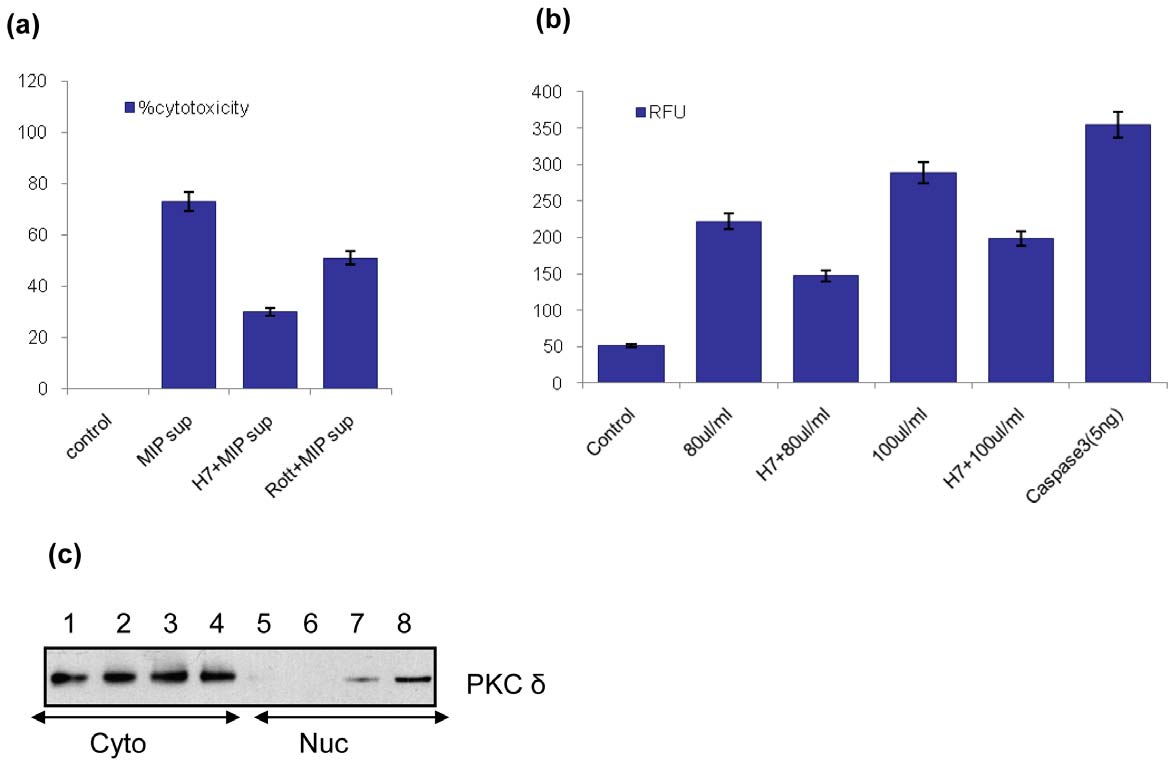
Involvement of PKC in MIP supernatant induced cell death of macrophages. (a) Macrophage monolayers were pretreated with H7 or Rottlerin for 45 min, incubated with MIP supernatant for 8 hr and % cytotoxicity was determined by MTT assay. Untreated macrophages were taken as control with 0% cytotoxicity. (b) Macrophages monolayers were pretreated with H7, incubated with different doses of MIP supernatant & activated caspase 3 was quantified using caspase3 FL detection kit. Inhibiting PKC also resulted in decreased caspase 3 activities. RFU: Relative Fluorescence Units. (c) MIP supernatant induced nuclear translocation of PKC δ. Macrophage monolayers were treated with MIP supernatant for 1, 2 or 3 hr. Cytoplasmic and nuclear fractions were harvested and immunoblotted for PKC δ. Lanes 1–4 (Cyto): control, 1 hr, 2 hr, 3 hr; lanes 5–8 (Nuc): control, 1 hr, 2 hr, 3 hr. Cyto-cytoplasmic fraction, Nuc-nuclear fraction.

Pretreatment of macrophages with H7, a broad range inhibitor of PKC and rottlerin, a pharmacological inhibitor of PKC δ inhibit MIP supernatant induced apoptosis significantly. Macrophages pretreated with H7 or rottlerin (10 µM) were incubated with MIP supernatant for 8 hr and % cytotoxicity was measured; H7 was found to inhibit apoptosis in as many as 60–70% of cells while rottlerin rescued ∼50% cells (Fig. 5a). Pretreatment of macrophages with H7 also resulted into decreased caspase 3 activity (Fig. 5b).

### Endogenous PKC δ translocates to the nucleus after treatment with MIP cell-free supernatant

PKC δ plays a crucial role in apoptosis induced by agents that target the mitochondria-dependent pathway and its nuclear translocation has been implicated in the process. To determine whether PKC δ translocates to the nucleus in apoptotic cells, nuclear and cytosolic fractions were separated and immunoblotted for PKC δ. Treatment with MIP supernatant led to a time dependent nuclear translocation of PKC δ (Fig. 5c).

### MIP supernatant downregulates LPS induced proinflammatory responses

**Figure 6:**
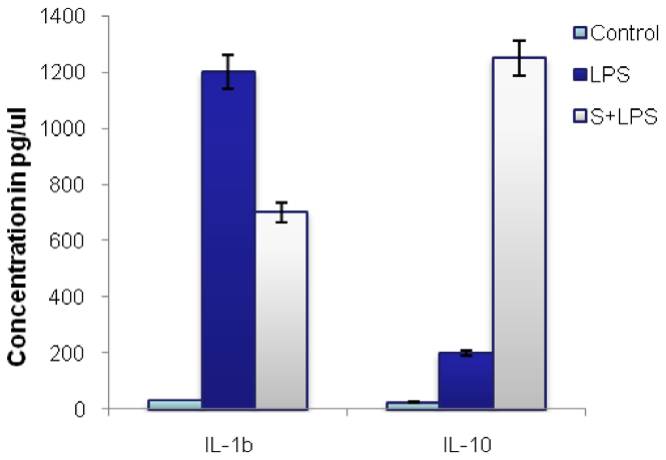
MIP Supernatant resulted into downregulation of LPS induced proinflammatory responses. Macrophage monolayers were treated with LPS without or in the presence of 40 ul of MIP supernatant for 12 hrs, supernatant was collected & ELISA was performed for IL-1β & IL-10. Supernatant led to enhanced expression of IL-10.

LPS has been shown to induce higher level-expression of proinflammatory cytokines in mouse peritoneal macrophages. The effect of MIP supernatant on LPS induced proinflammatory cytokines was observed. When co-incubated with MIP supernatant, LPS induced IL-β expression was markedly reduced by 12hrs of treatment. MIP supernatant also led to an enhanced expression of the Th2 cytokine IL-10 (Fig. 6).

## Discussion

*Mycobacterium indicus pranii* (MIP) has been tested in a number of disease models and its immunomodulatory properties in leprosy are well documented [2]–[4], [9]. The failure of BCG to protect against the most prevalent form of tuberculosis, pulmonary TB, has led to a heightened demand for more effective alternative vaccine candidates. MIP has recently been evaluated where vaccination with heat-killed MIP induces protection against TB in animal models [5]. Currently, heat-killed MIP is commercially available as a vaccine named “Immuvac”.

We started our investigations with the aim to look for immunomodulatory properties of heat-killed MIP using mouse peritoneal macrophages. It was observed that the cell-free supernatant collected from whole-cell MIP suspension induced rapid and significant apoptosis in mouse peritoneal macrophages *in vitro*. Apoptosis is generally characterized by early expression of Phosphatidylserine on the cell surface, membrane blebbing, nuclear condensation & DNA fragmentation, and thus detecting the appearance of PS on the outer cell membrane is a sensitive means to confirm the apoptotic mode of cell death. Staining of MIP supernatant treated macrophages with annexin V-Cy3 confirmed that MIP supernatant induced cell death of macrophages is mediated through apoptosis. LPS contamination as the suspected cause of cell death was experimentally excluded. MIP supernatant consistently showed the presence of a protein band of ∼46 kDa in SDS-PAGE. MALDI based analysis of this band indicated a significant homology to peptide chain release factor of bacterial origin. Peptide Chain Release Factor 1 carries an Inhibitor of Apoptosis Protein (IAP) binding motif (IBM). IBM containing proteins are thought to act as pro-apoptotic factors which act by preventing repression or degradation of caspases and are known to play role in mitochondria-mediated apoptosis. The IBM present in these proteins quenches the anti-apoptotic potential of IAPs by directly binding to their BIR (Baculovirus IAP Repeat) domain and thus until recently was thought to signify as the core of their apoptogenic activity [17]. In all animal species, apoptosis is linked to the activation of caspases, a class of cysteine proteases that participates in the dismantling of essential cellular components. Under these assumptions an IAP-binding protein would, as a rule, act in a caspase dependent manner. Treatment of macrophages with MIP supernatant agreeably resulted in induction of caspase 3 activity, but pretreatment of macrophages with pan-caspase inhibitor, Z-VAD-fmk failed to inhibit MIP supernatant induced apoptosis. The caspase independent action of the supernatant protein therefore, would refute the analogy irrespective of its homology to an IAP-binding protein. To the rescue is a recent demonstration that IAP-binding proteins may not necessarily require an IBM for their apoptogenic potential and therefore their apoptotic function is not mediated only through an interaction with the IAPs [18]. This would mean that IAP binding is a secondary function of these proteins and they may route through other pathways of apoptosis induction where caspase activity is only auxiliary to their apoptogenic potential. Caspase independent nature of apoptosis in this case tempted us to look for involvement of mitochondria in the process.

In mammals, apart from the classical receptor based activation, caspases are also activated as the result of MMP, an event that marks the point of no return in the death process [19]. The onset of MMP is an abrupt event during apoptosis, leading to the release of a number of apoptogenic proteins normally found in the space between the inner and outer mitochondrial membranes (including cytochrome c, AIF, and others) [20], [21]. Depending upon the initial cell death stimulus there are two different ways in which caspases can contribute to cell death. First, the apoptotic signal directly leads to activation of caspases which in turn execute the process. Alternatively, caspases can be activated at a later stage of the process after MMP. In this case caspase activation appears to constitute a sign rather than a mechanism of cell death and the decisive event has occurred upstream or at the level of MMP, though even later activation of caspase 3 can accelerate the kinetics of apoptosis [19], [22]. A significant population of MIP supernatant treated macrophages showed decreased Δψ_m_ by 4 hr of treatment indicating MMP as the major factor regulating supernatant induced apoptosis of macrophages. Apparently, in our model caspase activation and mitochondrial alteration are either mutually exclusive events or the activation of caspases is a post-mitochondrial outcome.

Bcl-2 family member Bax has been shown to play a major role in MMP. The mere addition of Bax to isolated mitochondria may lead to release of pro-apoptotic proteins. Conversely, Bax deficient cells may show complete resistance towards apoptotic stimuli implicating Bax as one of the earliest determinants of cellular apoptotic program. In most of the cells Bax is located predominantly in the cytosol, but translocates to mitochondria at an early stage in apoptosis [23], [24]. During early hours of supernatant treatment, a marked translocation of Bax onto the mitochondria is observed. It is proposed that MIP supernatant leads to translocation of cytoplasmic Bax to the mitochondria and thus induces MMP. Furthermore, MIP supernatant induced MMP and subsequent release of AIF and cytochrome c into the cytoplasm and further translocation of AIF into the nucleus, as observed, may be the key discrete events responsible for the execution of programmed cell death of mouse peritoneal macrophages. Experiments with pharmacological inhibitors revealed that the supernatant induced Bax translocation and thus MMP may be assisted by the members of PKC family as PKC inhibitors inhibited macrophage apoptosis to a great extent in our model. Recently the nuclear accumulation of PKC δ has been documented as a prerequisite for apoptosis in some cell types and evidences suggest that nuclear PKC δ regulates the cytosolic apoptotic machinery acting as a regulator of mitochondria-dependent apoptosis [25]–[28]. To look for this possibility, nuclear and cytoplasmic fractions were harvested from MIP supernatant treated macrophages and probed for the presence of PKC δ. Appreciable amount of nuclear PKC δ was detected by as early as 2 hr of treatment. The kinetics of mitochondrial translocation of Bax paralleled the nuclear translocation of PKC δ suggesting some kind of cooperativity between these two events, though it was hard to find a direct relation. Moreover, a number of nuclear proteins have been characterized to be substrates of PKC δ *in vitro* [29]. We report here that the novel protein kinase C isoform, PKC δ, is required at or prior to the level of the mitochondria for MIP supernatant induced apoptosis of macrophages. With AIF getting translocated to the nucleus along with PKC δ, MIP supernatant probably activated two discrete, although seemingly dependent mechanisms of apoptosis.

In recent times, there has been considerable interest in understanding how microbial invasion may affect apoptosis of eukaryotic cells and whether this contributes to pathogenesis. For example, *M. tuberculosis* and other mycobacteria as well as a variety of unrelated microbial pathogens have been reported to induce apoptosis of host cells [30]–[32]. In some instances, apoptosis has been observed to occur after infection of cells with viable organisms and in other cases upon incubation of cells with subcellular bacillary and secretory factors [33]–[37]. A repertoire of mycobacterial products, including culture filtrate of *M. tuberculosis*, supernatant from heat-treated mycobacteria, and purified protein derivative have been shown to induce apoptosis. Apoptosis as a phenomenon is associated with lack of inflammatory responses in general but may also be associated with suppression of pro-inflammatory cytokine production as in the case of Mycolactone, a secretory product of *Mycobacterium ulcerans* [38]. Unlike washed MIP cell pellet, sub-threshold doses of MIP supernatant led to a significant downregulation of LPS induced expression of proinflammatory cytokine IL-1β. The observed drop in the levels of IL-1β may be a direct consequence of the elevated levels of Th2 cytokine IL-10. Simultaneous treatment of peritoneal macrophages with MIP supernatant led to increment in the expression of IL-10 by several folds. The present study points toward the fact that concomitant induction of IL-10 by MIP supernatant may downsize the overall effective Th1 response generated by washed MIP cell pellet. Furthermore, the apoptogenic property of MIP supernatant may also account for some of the untoward effects of the vaccine at the site of administration [2]. Under such conditions the supernatant component of the original MIP suspension could be a heavy compromise to the immunomodulatory potential of heat-killed MIP as a vaccine. In the background of MIP generating substantial success as a vaccine, not only prophylactic, but also therapeutic, the current study may bear some critical implications and needs immediate attention.
